# Effects of Environmental pH on the Growth of Gastric Cancer Cells

**DOI:** 10.1155/2020/3245359

**Published:** 2020-03-09

**Authors:** Wenjie Li, Ying Zhou, Chunyu Shang, Hui Sang, Hong Zhu

**Affiliations:** Department of Gastroenterology, The First Affiliated Hospital of Nanjing Medical University, 300 Guangzhou Road, Nanjing 210029, China

## Abstract

**Background:**

Proton pump inhibitor (PPI) and other acid-suppressing drugs are widely used in the treatment of gastrointestinal ulcer, upper gastrointestinal bleeding, gastritis, and gastric cancer (GC). About 80% of GC patients receive acid suppression treatment. PPI suppresses the production of gastric acid by inhibiting the function of H^+^/K^+^-ATPase in gastric parietal cells and raises the pH value to achieve therapeutic purposes. Some studies have found that PPI had a certain antitumor effect in the proliferation and apoptosis of tumor cells. But the effects of environmental pH on the growth of GC cells and its mechanism are unknown. Therefore, we hoped to find the effects of culture medium pH on the biological behavior of GC cells by in vitro experiments and provide guidance for the use of acid-suppressing drugs in GC patients.

**Aims:**

We aimed to observe the effects of pH changes in GC cell culture medium on the cell biological behavior of cancer cells and to analyze the potential mechanisms. We hoped to find out the effect of acid suppression on the growth of GC cells.

**Methods:**

The GC cell lines (SGC-7901 and MKN45) were used as the research object. We adjusted the pH value in the cell culture medium to observe the changes in cell viability (MTT), apoptosis (flow cytometry), and invasion (Transwell) at pH 6, pH 7, and pH 8. qRT-PCR and western blot (WB) assays were used to determine the expression changes of genes and proteins (mTOR, AKT, Wnt, Glut, and HIF-1*α*) at pH 6, pH 7, and pH 8.

**Results:**

The results of MTT showed that the viability of SGC-7901 and MKN45 in the pH 8.0 group was significantly weaker than that in the pH 6.0 or pH 7.0 group (*P* < 0.001). Flow cytometry results showed that the apoptosis of SGC-7901 and MKN45 in the pH 8.0 group was more obvious than that in the pH 6.0 or pH 7.0 group (*P* < 0.001). Flow cytometry results showed that the apoptosis of SGC-7901 and MKN45 in the pH 8.0 group was more obvious than that in the pH 6.0 or pH 7.0 group (*P* < 0.001). Flow cytometry results showed that the apoptosis of SGC-7901 and MKN45 in the pH 8.0 group was more obvious than that in the pH 6.0 or pH 7.0 group (*α*) at pH 6, pH 7, and pH 8. *P* < 0.001). Flow cytometry results showed that the apoptosis of SGC-7901 and MKN45 in the pH 8.0 group was more obvious than that in the pH 6.0 or pH 7.0 group (

**Conclusions:**

Compared with the microacid environment, the microalkaline environment inhibited the viability, invasion, and expression of genes and proteins (mTOR, AKT, Wnt, Glut, and HIF-1*α*) but promoted the apoptosis of GC cells and thus inhibited the growth of GC.*α*) at pH 6, pH 7, and pH 8.

## 1. Introduction

Gastric cancer (GC) is the most common malignant tumor in the world, and its mortality ranks second among the malignant tumors [1]. In China, the incidence and mortality of GC are second only to lung cancer. At present, surgery-based, chemotherapy-assisted comprehensive therapy is widely used to treat GC. During hospitalization, most patients receive antiacid treatment with PPI and other drugs to relieve clinical symptoms and prevent gastrointestinal bleeding. Drugs, such as PPI, can suppress the production of gastric acid by inhibiting the function of H^+^/K^+^-ATPase in gastric parietal cells and thus reduce gastric acid secretion and raise the pH value to achieve therapeutic purposes [2]. Some researchers believed that PPI had antitumor effects [3–5], while other studies suggested that PPI might increase the risk of GC [6, 7]. Under pathological conditions, GC tissues and cells are in an environment of gastric juice. It is not clear whether the change of gastric acid concentration in gastric juice could affect the growth of gastric cancer cells. In this study, we investigated the effects of pH changes in GC cell culture medium on the biological behavior of GC cells by in vitro experiments. We expected to find out the effect of acid suppression on GC growth.

## 2. Materials and Methods

### 2.1. Cell Culture

Human GC cell lines SGC-7901 and MKN45 were provided by the Laboratory of Department of Gastroenterology, the First Affiliated Hospital of Nanjing Medical University (Nanjing, Jiangsu, China). The cells were cultured in RPMI-1640 medium with 10% FBS and penicillin-streptomycin at 37°C in an atmosphere containing 5% CO_2_.

### 2.2. Experimental Groups

In each of the following experiments, the pH of the GC cell culture medium was adjusted by sodium bicarbonate, and the pH value of the culture solution was monitored by a pH meter. The experiments were divided into three groups in which the pH was maintained at pH 6.0, pH 7.0, and pH 8.0, respectively, and pH 7.0 was set in the control group.

### 2.3. Reagents and Instruments

RPMI-1640 was purchased from GIBCO (USA). Penicillin-streptomycin and 0.25% Trypsin-EDTA were purchased from Jiangsu Kaiji Biotechnology Co., Ltd. (Jiangsu, China). Calf serum was purchased from Hangzhou Sijiqing Biological Engineering Materials (Zhejiang, China). Fetal bovine serum was purchased from ExCell Biology (USA). TRIzol was purchased from Invitrogen (USA). WB wash buffer, WB blocking buffer, WB primary antibody diluent, WB second antibody dilution, and WB stripping buffer were purchased from Nanjing Lufei Biotechnology Co., Ltd. (Jiangsu, China). *β*-Actin was purchased from ImmunoWay (USA). Goat anti-mouse IgG/HRP and goat anti-rabbit IgG/HRP were purchased from Jackson (USA). Antibodies against Glut1 (1 : 1000; 12939), AKT (1 : 1000; 9272), mTOR (1 : 1000; 2983), HIF-1*α* (1 : 1000; 14179), and *β*-actin (1 : 1000; 3700) were purchased from Cell Signaling Technology (Danvers, MA, USA), and antibody for wnt3a (1 : 1000; ab234099) was purchased from Abcam (Cambridge, UK).

### 2.4. Cell Viability Analysis

MTT assay was used to evaluate GC cell viability according to the manufacturer's instructions. GC cells were placed in 96-well plates at a density of 3~5 × 10^5^ cells per well. The plates were incubated at 37°C in an atmosphere containing 5% CO_2_ for 24 h. The drug was diluted to the desired concentration with complete medium. Then, 100 *μ*L drug-containing medium was added to each well. A negative control group and a positive control group were set up. The plates were incubated at 37°C in an atmosphere containing 5% CO_2_ for 72 h. Next, 20 *μ*L MTT solution was added. 4 h later, 150 *μ*L DMSO solution was added and the optical density was 490 nm as shown by a microplate reader.

### 2.5. Cell Apoptosis Analysis

To detect the apoptosis of the GC cells, the cultured SGC-7901, and MKN45 in the logarithmic growth phase were digested with 0.25% trypsin. GC cells were washed with PBS and stained with Annexin V together with propidium iodide for 15 min at room temperature in the dark as recommended by the manufacturer. Then, the apoptosis of GC cells was analyzed by a flow cytometer.

### 2.6. Cell Invasion Analysis

Transwell invasion assay was used to determine the invasion ability of the cells according to the manufacturer's instructions. The assays on cell invasion ability were performed in 24-well Transwell chambers, which were coated with Matrigel. SGC-7901 and MKN45 were first treated with 0.25% trypsin and then hung in incomplete medium for 24 h. The cell density was adjusted to 5 × 10^5^ cells/mL. Then, 100 *μ*L cell suspension was added to the Transwell chamber, whereas the lower chamber was filled with 500 *μ*L of medium supplemented with 20% fetal bovine serum (FBS). The cells were then incubated at 37°C in an atmosphere containing 5% CO_2_ and 1% O_2_. The cells in the upper chamber were wiped with a cotton swab and stained with 0.1% crystal violet for 30 min. Finally, cell numbers were counted under a microscope at a magnification of ×200.

### 2.7. qRT-PCR Analysis

Total RNA was extracted from GC cells using the TRIzol reagent (Invitrogen) according to the manufacturer's instructions. Subsequently, they were reversely transcribed to obtain cDNA using the First Strand cDNA Synthesis Kit. qRT-PCR was conducted using the SYBR Green qPCR Master Mix based on the manufacturer's protocol. Primers were designed and synthesized by Nanjing Jinsrui Technology Co., Ltd. (Nanjing, China) (human-GAPDH primer (132 bp): sense primer: 5-TGGTATCGTGGAAGGACTCA-3, antisense primer: 5-CCAGTAGAGGCAGGGATGAT-3; human-mTOR primer (130 bp), sense primer: 5-ATTCCGACCTTCTGCCTTCA-3, antisense primer: 5-CACAGCCACAGAAAGTAGCC-3; human-AKT primer (136 bp), sense primer: 5-GCGTGACCATGAACGAGTTT-3, antisense primer: 5-TTGGCCACGATGACTTCCTT-3; human-Wnt primer (144 bp), sense primer: 5-TTCTCCCAGTCTCTGTCGTG-3, antisense primer: 5-CATCCAAACTCGTGGCTCTG-3; human-Glut primer (126 bp), sense primer: 5-GGGCATGTGCTTCCAGTATG-3, antisense primer: 5-AAGGTCCGGCCTTTAGTCTC-3; and human-HIF-1*α* primer (142 bp), sense primer: 5-TGCTGATTTGTGAACCCATT-3, antisense primer: 5-TCTGGCTCATATCCCATCAA-3). Relative gene expression levels were detected and calculated using the 2^-*ΔΔ*Ct^ comparative method.

### 2.8. WB Analysis

WB was used to detect the expression of mTOR, AKT, Wnt, Glut, and HIF-1*α* proteins. Protein extraction in each group was performed according to the protein extraction steps. The protein concentration was estimated using the BCA Protein Assay Kit. Subsequently, protein was electrophoresed in sodium dodecyl sulfate-polyacrylamide gel electrophoresis gels and transferred to PVDF membranes. Blots were blocked with 5% skim milk for 1 h and incubated overnight with the primary antibody at 4°C. The next day, the membranes were incubated with the corresponding IgG–HRP secondary antibody (1 : 5000) for 1-2 h at room temperature. Finally, the exposure was developed using a developing mixture, and the signals were normalized using *β*-actin control.

### 2.9. Statistical Analysis

The data were presented as mean + standard deviation (SD). SPSS 22.0 software (SPSS, Chicago, IL, USA) was used for statistical analyses. Significant differences between groups were assessed using GraphPad Prism 5 software (San Diego, CA, USA). The groups were compared using one-way analysis of variance (ANOVA), and the LSD method was used to determine the differences between groups. *P* < 0.05 was considered statistically significant, and ns meant *P* > 0.05, ∗ meant *P* < 0.05, ∗∗ meant *P* < 0.01, and ∗∗∗ meant *P* < 0.001.

## 3. Results and Conclusions

### 3.1. Alkaline Microenvironment Inhibited Viability of GC Cells

**Figure 1:**
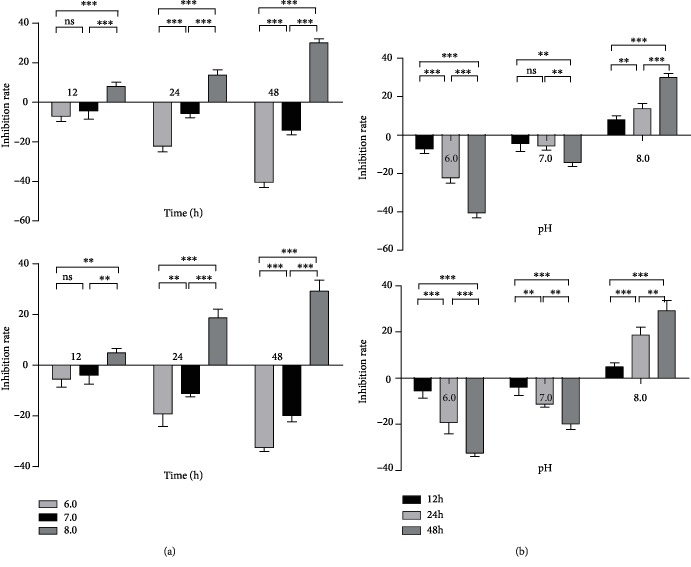
Alkaline microenvironment inhibited the viability of GC cells. Compared with the blank control group, the optical density (OD) value increased in the pH 6.0 or pH 7.0 group, and the inhibition rate was negative, indicating cell promotion. When the OD value of the pH 8.0 group was lower than that of the blank group, the inhibition rate was positive, indicating cells inhibition. When pH 6.0 or pH 7.0, the viability of GC cells was promoted. The smaller the pH value was, the smaller the inhibition rate of cells was, and the greater the viability of cells was. However, in the pH 8.0 group, the viability of GC cells was inhibited, showing that the alkaline environment inhibited the viability of GC cells (b). (a) Viability analysis of GC cells (SGC-7901 and MKN45) treated with different culture times (12 h, 24 h, and 48 h). (b) Viability analysis of GC cells (SGC-7901 and MKN45) treated with culture media of different pH values (pH 6.0, pH 7.0, and pH 8.0).

MTT assay was used to detect the viability of SGC-7901 and MKN45 at pH 6.0, pH 7.0, and pH 8.0, respectively. We found that SGC-7901 and MKN45 had no significant difference in viability rate between pH 6.0 and pH 7.0 after being cultured for 12 h (*P* > 0.05). Compared with the pH 6.0 or pH 7.0 group, the cell viability rate of the pH 8.0 group decreased (*P* < 0.01). There were significant differences in cell viability between pH 6.0 vs. pH 7.0, pH 7.0 vs. pH 8.0, and pH 6.0 vs. pH 8.0 (*P* < 0.01) after 24 h or 48 h of culturing. We also found that the differences in cell viability of GC cells between pH 6.0 vs. pH 7.0, pH 7.0 vs. pH 8.0, and pH 6.0 vs. pH 8.0 were the most obvious after being cultured for 48 h (*P* < 0.001) (Figure 1(a)).

### 3.2. Alkaline Microenvironment Promoted Apoptosis of GC Cells

**Figure 2:**
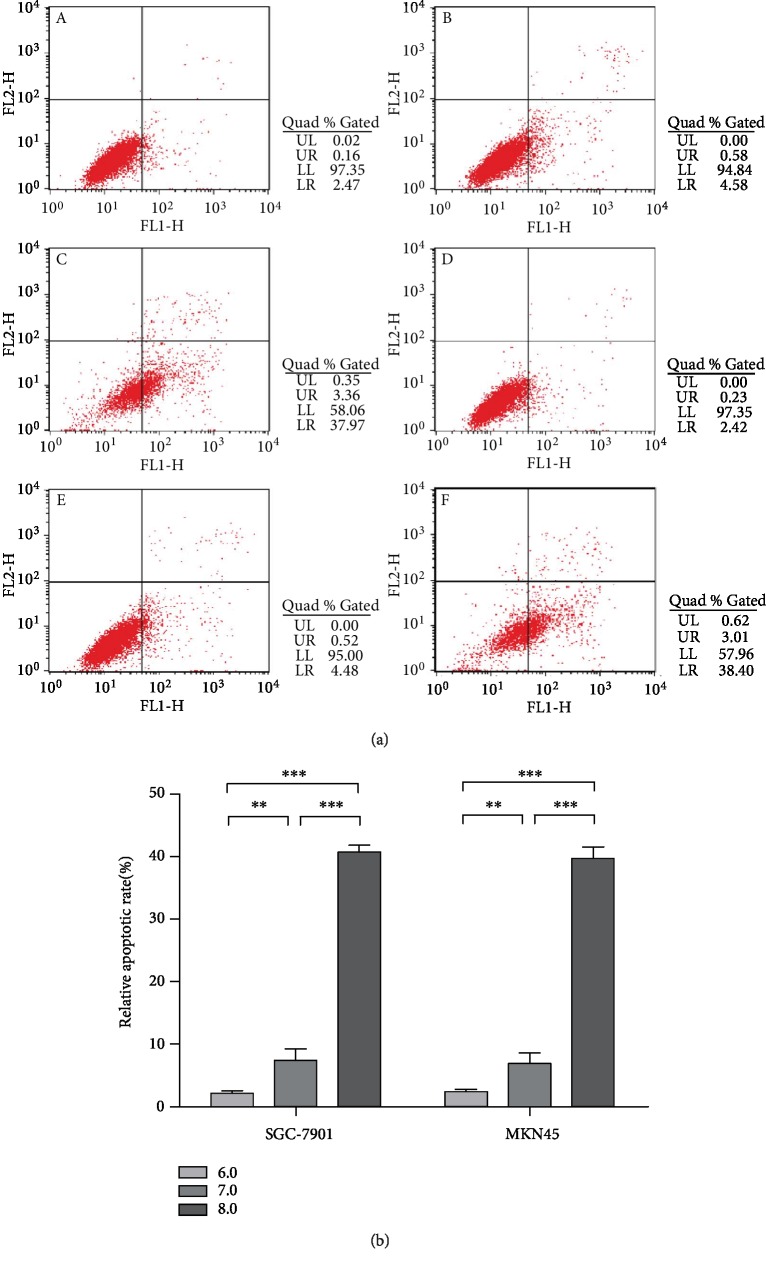
Alkaline microenvironment promoted the apoptosis of GC cells. (a) Apoptosis of GC cells at different pH values (pH 6.0, pH 7.0, and pH 8.0) after being cultured for 48 h. A, B, and C in (a), respectively, represented apoptosis of SGC-7901 cultured in a medium with pH 6.0, pH 7.0, and pH 8.0 for 48 h, respectively; D, E, and F represented apoptosis of MKN45 cultured in a medium at pH 6.0, pH 7.0, and pH 8.0 for 48 h, respectively. (b) Apoptosis results of GC cells (SGC-7901 and MKN45) at media of different pH values (pH 6.0, pH 7.0, and pH 8.0) after being cultured for 48 h.

Flow cytometry was used to detect the apoptosis of GC cells (SGC-7901 and MKN45). The results showed that after treatment with different pH media for 48 h, GC cells showed different degrees of apoptosis, which was mainly early apoptosis (LR). Compared with the pH 6.0 group, the apoptosis rate of GC cells increased at pH 7.0 (*P* < 0.01). Compared with pH 7.0, the apoptosis rate of GC cells increased significantly at pH 8.0 (*P* < 0.001). Compared with pH 6.0, the apoptosis rate of GC cells increased significantly at pH 8.0 (*P* < 0.001). The results showed that with the increase of pH, the apoptotic rate of GC cells increased gradually, and the alkaline environment promoted apoptosis and inhibited tumor progression (Figure 2(b)).

### 3.3. Alkaline Microenvironment Inhibited Invasion of GC Cells

**Figure 3:**
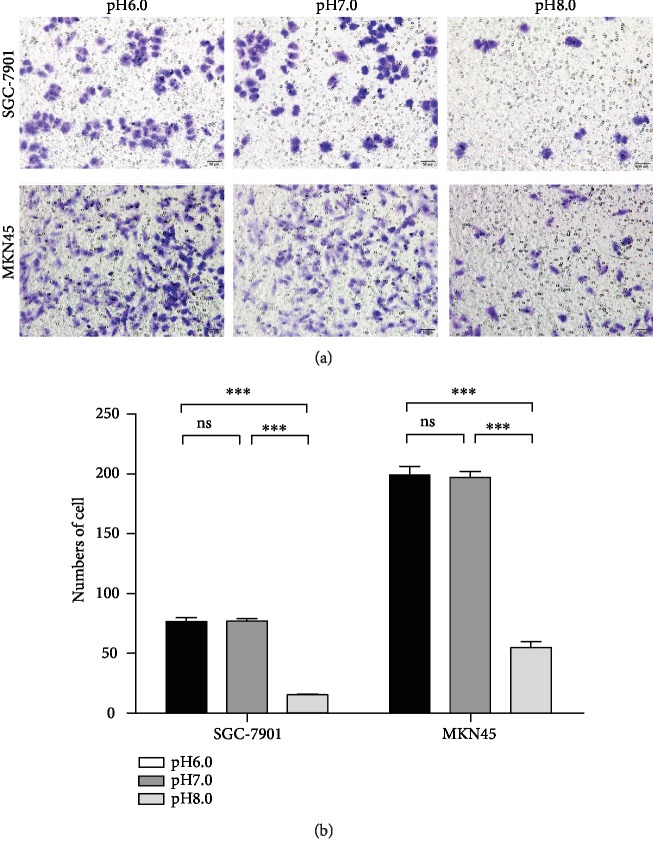
Alkaline microenvironment inhibited invasion of GC cells. (a) Micrograph of invasion of GC cells at different pH values (pH 6.0, pH 7.0, and pH 8.0) after being cultured for 48 h. (b) Analysis of invasive ability of GC cells at different pH values (pH 6.0, pH 7.0, and pH 8.0) after being cultured for 48 h.

The invasion ability of tumor cells was closely related to tumor metastasis. Transwell invasion assay results showed that no significant differences in the number of GC cells passing through Matrigel (mature gel) at pH 7.0 compared with pH 6.0 (*P* > 0.05); compared with pH 7.0, the number of GC cells passing through Matrigel at pH 8.0 was significantly different (*P* < 0.001). Compared with pH 6.0, the number of GC cells passing through Matrigel at pH 8.0 was significantly different (*P* < 0.001). This indicated that with the increase of pH, the invasion ability of GC cells decreased, and the alkaline environment inhibited the invasion of GC cells and tumor progression (Figure 3(b)).

### 3.4. Alkaline Microenvironment Inhibited the Expression of Cancer-Associated Genes and Proteins (mTOR, AKT, HIF-1*α*, Wnt, and Glut)

**Figure 4:**
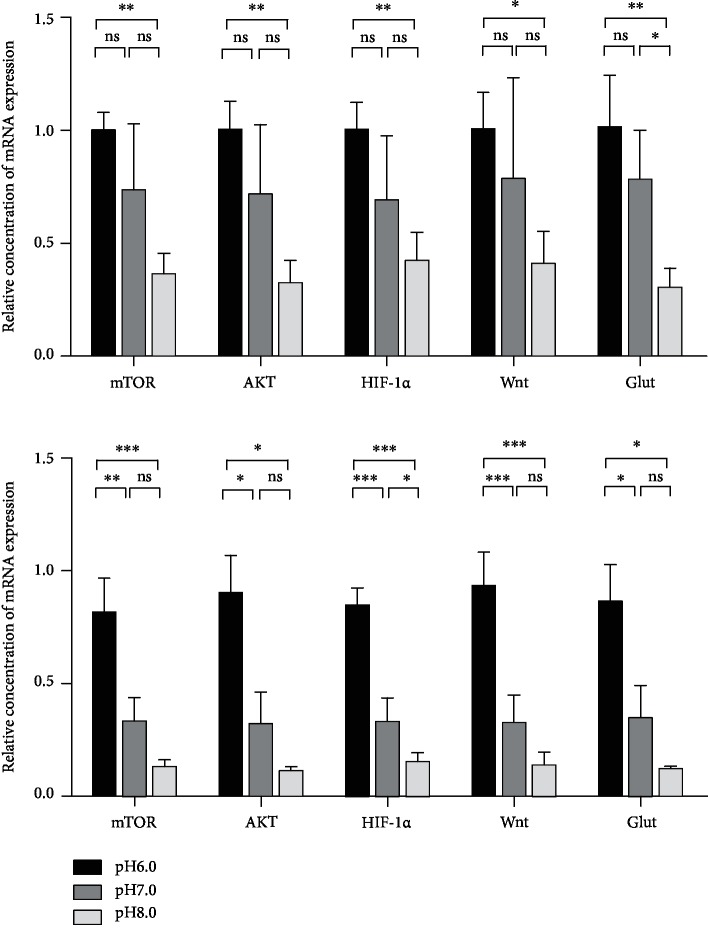
Alkaline microenvironment inhibited the expression of genes (mTOR, AKT, HIF-1*α*, Wnt, and Glut). (a) qRT-PCR detected the expression of genes (mTOR, AKT, HIF-1*α*, Wnt, and Glut) in GC cells (SGC-7901 and MKN45) at different pH values (pH 6.0, pH 7.0, and pH 8.0) after being cultured for 48 h.

The expression of cancer-associated genes (mTOR, AKT, HIF-1*α*, Wnt, and Glut) in GC cells (SGC-7901 and MKN45) was detected at the gene level at pH 6.0, pH 7.0, and pH 8.0, respectively. qRT-PCR results showed no significant differences between the expression of genes (mTOR, AKT, HIF-1*α*, Wnt, and Glut) in SGC-7901 at pH 7.0 and that at pH 6.0 (*P* > 0.05). The expression of mTOR, AKT, HIF-1*α*, and Wnt in SGC-7901 at pH 8.0 was not different from that at pH 7.0 (*P* > 0.05), but the expression of Glut was different (*P* < 0.05). The expression of mTOR, AKT, HIF-1*α*, Wnt, and Glut in MKN45 was different at pH 7.0 from that at pH 6.0 (*P* < 0.05). The expression of mTOR, AKT, Wnt, and Glut in MKN45 at pH 8.0 was not different from that at pH 7.0 (*P* > 0.05); however, the expression of HIF-1*α* was different (*P* < 0.05). In GC cells (SGC-7901 and MKN45), the expression of mTOR, AKT, HIF-1*α*, Wnt, and Glut at pH 8.0 was different from that at pH 6.0 (*P* < 0.05). This indicated that with the increase of pH, the expression of genes (mTOR, AKT, Wnt, Glut, and HIF-1*α*) in GC cells (SGC-7901 and MKN45) decreased, and the alkaline environment inhibited the gene expression in GC cells (Figure 4).

**Figure 5:**
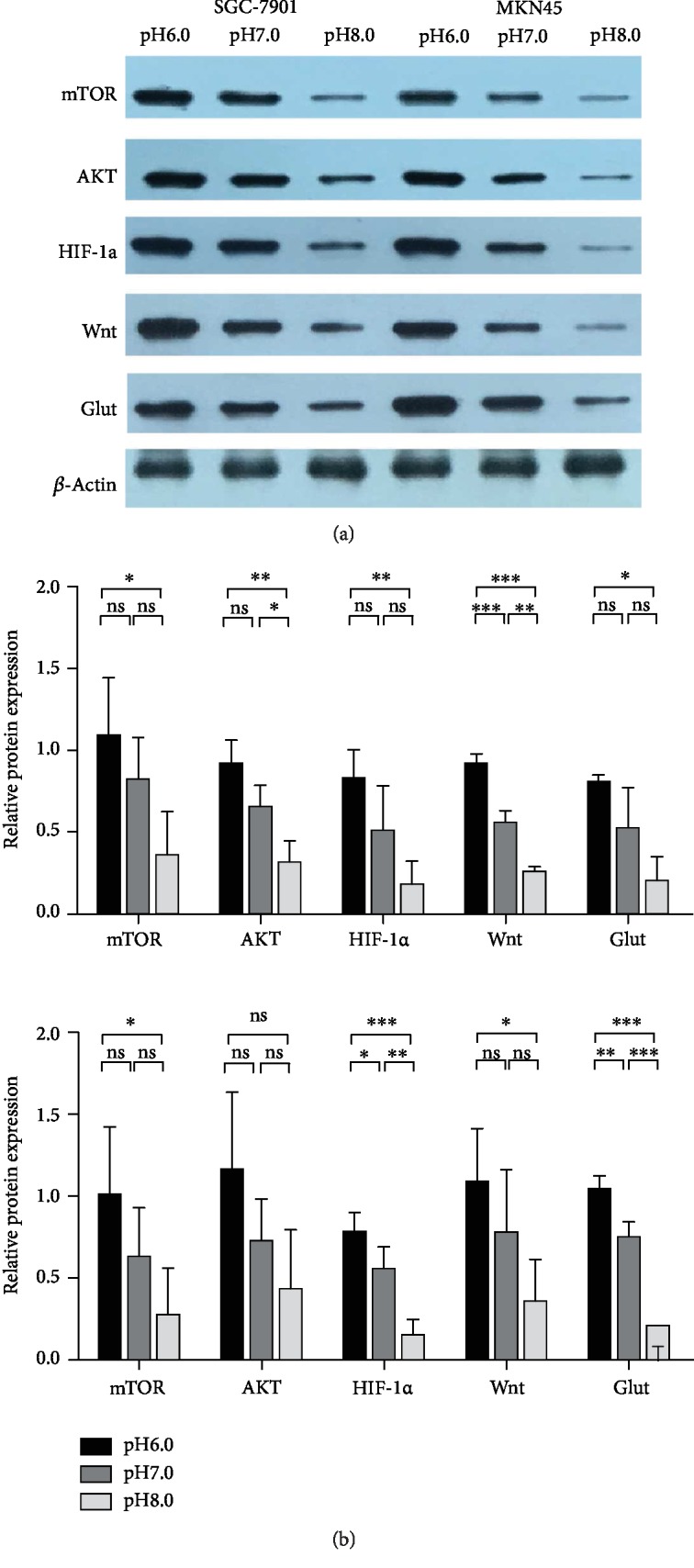
Alkaline microenvironment inhibited the expression of proteins (mTOR, AKT, HIF-1*α*, Wnt, and Glut). (a) Grayscale analysis of WB bands. (b) WB detected the expression of proteins (mTOR, AKT, HIF-1*α*, Wnt, and Glut) in GC cells (SGC-7901 and MKN45) at different pH values (pH 6.0, pH 7.0, and pH 8.0) after being cultured for 48 h.

WB results showed that there were no significant differences between the expression of mTOR, AKT, HIF-1*α*, and Glut proteins in SGC-7901 at pH 7.0 and that at pH 6.0 (*P* > 0.05), but the Wnt protein was significantly different (*P* < 0.001). The expression of mTOR, HIF-1*α*, and Glut proteins in SGC-7901 at pH 8.0 was not different from that at pH 7.0 (*P* > 0.05); however, the AKT and Wnt proteins were different (*P* < 0.05). The expression of mTOR, AKT, HIF-1*α*, Wnt, and Glut proteins in SGC-7901 at pH 8.0 was different from that at pH 6.0 (*P* < 0.05). The expression of HIF-1*α* and Glut proteins in MKN45 at pH 7.0 was different from that at pH 6.0 (*P* < 0.05), but the expression of mTOR, AKT, and Wnt proteins was not different (*P* > 0.05). The expression of HIF-1*α* and Glut proteins in MKN45 at pH 8.0 was significantly different from that at pH 7.0 (*P* < 0.01), but the expression of mTOR, AKT, and Wnt proteins was not different (*P* > 0.05). The expression of mTOR, HIF-1*α*, Wnt, and Glut proteins in MKN45 at pH 8.0 was significantly different from that at pH 6.0 (*P* < 0.01), but the expression of AKT was not different (*P* > 0.05). With an increase of pH, the expression of proteins (mTOR, AKT, Wnt, Glut, and HIF-1*α*) in GC cells (SGC-7901 and MKN45) decreased, but the AKT protein was not different in MKN45 at pH 6.0 vs. pH 8.0 (*P* > 0.05) (Figure 5(b)).

## 4. Discussion

A weak acid environment, which is common in solid tumor tissues, enhances the viability and invasion of tumor cells. The acidic microenvironment may be related to the “Warburg” effect of tumor cells, that is, under aerobic or anoxic conditions, the cells initiate glycolysis to convert glucose into lactic acid, a process in which energy is produced [8]. Then, the lactic acid accumulates in and around cells. The mild acidosis state of tumor cells has been shown to trigger the early stages of apoptosis, leading to DNA break by activating endonucleases. In order to avoid intracellular acidification, the expression of pH-regulated proteins in tumor cells is upregulated [9]. For instance, V-ATPase (vacuolar ATPase) pumps H^+^ out of the cell by the proton pump, forming an acid-base gradient, that is, the extracellular pH is acidic and the intracellular pH is alkaline [10]. In addition, the expression of Na^+^-H^+^ exchanger 1 (NHE-1) increases, and the excessive H^+^ in the cells is eliminated by Na^+^-H^+^ exchange. The expression of anion exchanger-2 (AE-2) reduces, and the Cl^−^/HCO3^−^ exchange is inhibited, which eliminates excessive HCO_3_^−^ in the cells and thereby promotes the formation of pH gradient [11, 12]. However, the mechanism of how this pH gradient is formed is still unclear. Several studies have confirmed that the acidic microenvironment can promote tumor progression by inducing genomic instability, promoting local invasion and migration, inhibiting antitumor immunity, and enhancing resistance to chemotherapeutic drugs [13–15].

In this study, MTT assay was used to compare the cell viability of GC cells SGC-7901 and MKN45 under different pH conditions. The results showed the viability activity of GC cells at pH 8.0 was significantly lower than that at pH 6.0 (*P* < 0.001) (Figure 1). Flow cytometry was used to compare the cell apoptosis rate of SGC-7901 and MKN45 under different pH conditions. The results showed the apoptosis rate of GC cells at pH 8.0 was significantly higher than that at pH 6.0 (*P* < 0.001) (Figure 2). Xu et al. [16] found that the changes of extracellular pH induced significant and rapid changes in autophagy activity, which was inhibited under acidic conditions. Since autophagy might be related to cell death [17], the decreased apoptosis rate of GC cells in an acidic environment might be related to the inhibition of autophagy activity. Qin et al. found that the autophagy inhibition of GC cells promoted epithelial-mesenchymal transition (EMT) and metastasis, and the metabolic type was also changed from mitochondrial oxidative phosphorylation to aerobic glycolysis [18]. EMT promotes the migration, invasion, and antiapoptosis of tumor cells, which in turn promotes the progression of GC cells [19]. In the future study, we will study the exact mechanism and focus on the expression of apoptosis-related genes such as Bax and Bcl-2 and EMT-related proteins such as E-cadherin, vimentin, and *β*-catenin.

In this study, Transwell was used to observe the invasion of SGC-7901 and MKN45 at different pH levels. The results showed that the invasive ability of GC cells at pH 6.0 was significantly higher than that at pH 8.0 (*P* < 0.001) (Figure 3). The results were consistent with the hypothesis of “acid-mediated tumor invasion.” Tumors export acid to the surrounding tissues, which stimulates the release of proteolytic enzymes by increasing the lysosomal cycle, thereby inducing normal cell death and promoting degradation of extracellular matrix [20]. Abaza and Luqmani [21] found that extracellular low pH could cause the secretion and activation of major proteolytic enzymes, including MMP-2, MMP-9, tissue serine protease, and gelatinase, leading to the degradation of extracellular matrix, thereby promoting tumor invasion. Compared with normal cells, V-ATPase is overexpressed and more active in tumor cells, which is positively correlated with the invasion of tumor cells [22]. Chen et al. [23] reported that PPI could inhibit the efflux of H^+^ mediated by V-ATPase, thereby reversing the transmembrane pH gradient and enhancing the sensitivity of SGC-7901 gastric cancer cell tumor drugs. Shen et al. [24] found that V-ATPase participated in Wnt/*β*-catenin signaling pathway, which promoted the development and metastasis of tumor, while PPI inhibited V-ATPase and blocked the pathway.

We detected the expression of mTOR, AKT, Wnt, Glut, and HIF-1*α* at the gene and protein levels in SGC-7901 and MKN45 at different pH by qRT-PCR and western blot, respectively, and tried to analyze the possible mechanism underlying the effect of environmental pH on GC cells. We found that at the gene expression level, the expressions of mTOR, AKT, HIF-1*α*, Wnt, and Glut at pH 8.0 were lower than that at pH 6.0. At the protein expression level, the AKT protein was not different in MKN45 at pH 6.0 vs. pH 8.0 (*P* > 0.05), and the expression trends of other proteins were consistent with the trend of gene expression. This might be a result bias caused by a small sample size. At pH 8.0, the protein expression of mTOR, Wnt, Glut, and HIF-1*α* was significantly inhibited (*P* < 0.05) (Figure 5). Therefore, we concluded that a weak alkaline microenvironment inhibited the expression of genes and proteins closely related to tumor progression in GC cells.

The adaptation of tumor cells to hypoxia and changes in glucose metabolism are the biological characteristics of tumor cells. Through the “Warburg” effect, tumor cells produce large amounts of lactic acid to form a weak acid environment, which is also a hallmark of hypoxic tissues and many solid tumors. Hypoxia in tumor tissues inhibits the degradation of hypoxia-inducible factor-1*α* (HIF-1*α*) and increases its local accumulation. HIF-1*α* is a key transcriptional regulator that mediates the adaptation of cells to hypoxic microenvironment and plays an important role in tumor cell energy metabolism, growth invasion, and angiogenesis [25–27]. HIF-1*α* induces angiogenesis and glycolysis by activating the expression of vascular endothelial growth factor (VEGF), glucose transporter (Glut), and glycolysis-related enzymes to control the transfer of oxygen and nutrients and improve the growth and proliferation of cancer cells. The HIF-1*α*-Glut pathway plays an important role in tumor progression [27–29].

The stability of HIF-1*α* is dependent on the phosphatidylinositol 3-kinase (PI3K)/AKT pathway [30], which regulates the transcriptional activity of extracellular signal-regulated kinase (ERK), suggesting that the increased expression and activity of HIF-1*α* may be related to Wnt/*β*-catenin signaling pathway. In gastrointestinal tumors, the Wnt signaling pathway is regulated by intracellular *β*-catenin level. A high level of *β*-catenin activates the Wnt pathway, which affects cell proliferation, differentiation, and microenvironmental adaptation [31]. HIF-1*α* competes with Tcf-4 for binding to *β*-catenin and activates a typical Wnt/*β*-catenin signaling pathway to adapt to hypoxia [32]. In addition, studies [33] suggested that HIF-1*α* might also regulate the invasiveness of cancer cells by altering the expression of intermediate filament (vimentin, keratin), extracellular matrix component (fibronectin), and protease (MMP2 and urokinase plasminogen activator receptor). The HIF-1*α* pathway plays an important role in tumor progression, but the specific mechanism of HIF-1*α* promoting tumor cell proliferation is not yet fully understood.

mTOR is a central regulatory kinase that induces the expression of related proteins in cell proliferation and metabolism. mTOR also promotes the expression of HIF-1*α*, thereby promoting the expression of VEGF and inducing angiogenesis. The mTOR signal of some growth factors regulates mTOR activity through the PI3K/Akt pathway [34]. Another mechanism of tumorigenesis driven by mTOR is through the inhibition of 4EBP1 by complex mTORC1, which activates eukaryotic translation initiation factor 4B (eIF4E), leading to the translation of cell cycle regulatory genes and oncogenes (such as antiapoptotic protein MCL-1) in the RNA and promoting the survival of cancer cells in mouse models [35]. Recent studies have shown that V-ATPase mediates the activation and autophagy of mTORC1, further demonstrating that mTORC1 localization on the surface of lysosomes is essential for its activation [36]. We speculated that alkaline microenvironment inhibited the intracellular P13K/Akt/mTOR signal pathway, so as to inhibit the downstream mTOR molecules, and then inhibit the expression of HIF-1*α*. In the next experimental study, we will further study the exact mechanism pathway to confirm the conjecture.

This study investigated the effects of different pH on GC cells. The results showed that alkaline microenvironment inhibited the viability and invasion of GC cells and the expression of mTOR, AKT, Wnt, Glut, and HIF-1*α* genes and proteins, promoted tumor cell apoptosis, and inhibited GC progression. Hopefully, these findings can provide guidance for the use of acid-suppressing drugs in GC patients, but the specific mechanism of pH affecting GC cells needs further study. There were still some defects in our study, for example, our data were obtained in vitro SGC-7901 and MKN45 cells, and an in vivo animal model were missing. In the future, we will do some research on nude mice transplanted tumor model and analyze the effects of different feeding solution pH value and PPI on gastric cancer transplanted tumor.

## Data Availability

The data used to support the findings of this study are available from the corresponding author upon request.

## Abbreviations

PPI: Proton pump inhibitor
FBS: Fetal bovine serum
GC: Gastric cancer
OD: Optical density
WB: Western blot
EMT: Epithelial-mesenchymal transition
VEGF: Vascular endothelial growth factor
Glut: Glucose transporter.

## References

[1] Yang Y., Bae W. K., Nam S. J. (2018). Acetonic extracts of the endolichenic fungus EL002332 isolated from *Endocarpon pusillum* exhibits anticancer activity in human gastric cancer cells. *Phytomedicine*.

[2] Reimer C. (2013). Safety of long-term PPI therapy. *Best Practice & Research. Clinical Gastroenterology*.

[3] Yeo M., Kim D. K., Kim Y. B. (2004). Selective induction of apoptosis with proton pump inhibitor in gastric cancer cells. *Clinical Cancer Research*.

[4] Luciani F., Spada M., de Milito A. (2004). Effect of proton pump inhibitor pretreatment on resistance of solid tumors to cytotoxic drugs. *Journal of the National Cancer Institute*.

[5] Lu Z. N., Tian B., Guo X. L. (2017). Repositioning of proton pump inhibitors in cancer therapy. *Cancer Chemotherapy and Pharmacology*.

[6] Waldum H. L., Fossmark R. (2017). Proton pump inhibitors and gastric cancer: a long expected side effect finally reported also in man. *Gut*.

[7] Ko Y., Tang J., Sanagapalli S., Kim B. S., Leong R. W. (2016). Safety of proton pump inhibitors and risk of gastric cancers: review of literature and pathophysiological mechanisms. *Expert Opinion on Drug Safety*.

[8] Westley R. L., May F. E. B. (2013). A twenty-first century cancer epidemic caused by obesity: the involvement of insulin, diabetes, and insulin-like growth factors. *International Journal of Endocrinology*.

[9] Izumi H., Torigoe T., Ishiguchi H. (2003). Cellular pH regulators: potentially promising molecular targets for cancer chemotherapy. *Cancer Treatment Reviews*.

[10] Katara G. K., Jaiswal M. K., Kulshrestha A., Kolli B., Gilman-Sachs A., Beaman K. D. (2014). Tumor-associated vacuolar ATPase subunit promotes tumorigenic characteristics in macrophages. *Oncogene*.

[11] Rotin D., Steele-Norwood D., Grinstein S., Tannock I. (1989). Requirement of the Na+/H+ exchanger for tumor growth. *Cancer Research*.

[12] Heming T. A., Bidani A. (2003). Intracellular pH regulation in U937 human monocytes: roles of V-ATPase and Na+/H+ exchange. *Immunobiology*.

[13] Fan S. H., Wang Y. Y., Wu Z. Y. (2015). AGPAT9 suppresses cell growth, invasion and metastasis by counteracting acidic tumor microenvironment through KLF4/LASS2/V-ATPase signaling pathway in breast cancer. *Oncotarget*.

[14] Song J., Ge Z., Yang X. (2015). Hepatic stellate cells activated by acidic tumor microenvironment promote the metastasis of hepatocellular carcinoma via osteopontin. *Cancer Letters*.

[15] Pilon-Thomas S., Kodumudi K. N., el-Kenawi A. E. (2016). Neutralization of tumor acidity improves antitumor responses to immunotherapy. *Cancer Research*.

[16] Xu T., Su H., Ganapathy S., Yuan Z. M. (2011). Modulation of autophagic activity by extracellular pH. *Autophagy*.

[17] Lum J. J., Bauer D. E., Kong M. (2005). Growth factor regulation of autophagy and cell survival in the absence of apoptosis. *Cell*.

[18] Qin W., Li C., Zheng W. (2015). Inhibition of autophagy promotes metastasis and glycolysis by inducing ROS in gastric cancer cells. *Oncotarget*.

[19] Miyata Y., Kanda S., Mitsunari K., Asai A., Sakai H. (2014). Heme oxygenase-1 expression is associated with tumor aggressiveness and outcomes in patients with bladder cancer: a correlation with smoking intensity. *Translational Research*.

[20] Ibrahim-Hashim A., Estrella V. (2019). Acidosis and cancer: from mechanism to neutralization. *Cancer Metastasis Reviews*.

[21] Abaza M., Luqmani Y. A. (2013). The influence of pH and hypoxia on tumor metastasis. *Expert Review of Anticancer Therapy*.

[22] De Milito A., Canese R., Marino M. L. (2010). pH-dependent antitumor activity of proton pump inhibitors against human melanoma is mediated by inhibition of tumor acidity. *International Journal of Cancer*.

[23] Chen M., Lu J., Wei W. (2018). Effects of proton pump inhibitors on reversing multidrug resistance via downregulating V-ATPases/PI3K/Akt/mTOR/HIF-1alpha signaling pathway through TSC1/2 complex and Rheb in human gastric adenocarcinoma cells in vitro and in vivo. *OncoTargets and Therapy*.

[24] Chen M., Zou X., Luo H. (2009). Effects and mechanisms of proton pump inhibitors as a novel chemosensitizer on human gastric adenocarcinoma (SGC7901) cells. *Cell Biology International*.

[25] Seagroves T. N., Ryan H. E., Lu H. (2001). Transcription factor HIF-1 is a necessary mediator of the pasteur effect in mammalian cells. *Molecular and Cellular Biology*.

[26] Ravi R., Mookerjee B., Bhujwalla Z. M. (2000). Regulation of tumor angiogenesis by p53-induced degradation of hypoxia-inducible factor 1*α*. *Genes & Development*.

[27] Kung A. L., Wang S., Klco J. M., Kaelin W. G., Livingston D. M. (2000). Suppression of tumor growth through disruption of hypoxia-inducible transcription. *Nature Medicine*.

[28] Urano N., Fujiwara Y., Doki Y. (2006). Overexpression of hypoxia-inducible factor-1 alpha in gastric adenocarcinoma. *Gastric Cancer*.

[29] Akakura N., Kobayashi M., Horiuchi I. (2001). Constitutive expression of hypoxia-inducible factor-1alpha renders pancreatic cancer cells resistant to apoptosis induced by hypoxia and nutrient deprivation. *Cancer Research*.

[30] Minet E., Michel G., Mottet D., Raes M., Michiels C. (2001). Transduction pathways involved in hypoxia-inducible factor-1 phosphorylation and activation. *Free Radical Biology & Medicine*.

[31] Liu H. L., Liu D., Ding G. R., Liao P. F., Zhang J. W. (2015). Hypoxia-inducible factor-1alpha and Wnt/beta-catenin signaling pathways promote the invasion of hypoxic gastric cancer cells. *Molecular Medicine Reports*.

[32] Kolligs F. T., Bommer G., Goke B. (2002). Wnt/beta-catenin/tcf signaling: a critical pathway in gastrointestinal tumorigenesis. *Digestion*.

[33] Isobe T., Aoyagi K., Koufuji K. (2013). Clinicopathological significance of hypoxia-inducible factor-1 alpha (HIF-1alpha) expression in gastric cancer. *International Journal of Clinical Oncology*.

[34] Al-Batran S. E., Ducreux M., Ohtsu A. (2012). mTOR as a therapeutic target in patients with gastric cancer. *International Journal of Cancer*.

[35] Fennelly C., Amaravadi R. K. (2017). Lysosomal biology in cancer. *Methods in Molecular Biology*.

[36] Meo-Evoli N., Almacellas E., Massucci F. A. (2015). V-ATPase: a master effector of E2F1-mediated lysosomal trafficking, mTORC1 activation and autophagy. *Oncotarget*.

